# TBL1XR1 mutation predicts poor outcome in primary testicular diffuse large B-cell lymphoma patients

**DOI:** 10.1186/s40364-020-00189-1

**Published:** 2020-04-17

**Authors:** Xinfeng Wang, Xiaoyu Xu, Wenzhi Cai, Haiyan Bao, Hongming Huang, Yifei Liu, Xi Yang, Changgeng Ruan, Depei Wu, Hongjie Shen, Suning Chen

**Affiliations:** 1 Jiangsu Institute of Hematology, Key Laboratory of Thrombosis and Hemostasis of Ministry of Health, the First Affiliated Hospital of Soochow University, National Center of Hematological Clinical Medicine Research, Shizi street 188, Suzhou, 215006 People’s Republic of China; 2 The First Affiliated Hospital of Nantong University, Nantong, People’s Republic of China; 3 Institute of Blood and Marrow Transplantation, Collaborative Innovation Center of Hematology, Soochow University, Suzhou, People’s Republic of China

**Keywords:** Primary testicular lymphoma, Gene mutation, TBL1XR1, Overall survival

## Abstract

Primary testicular lymphoma (PTL), often appearing as focal masses in the scrotum and epididymides, is the most frequent testicular tumor in aged men. Although MYD88 and CD79B mutations were the most common genetic alterations observed, the gene mutation landscape of PTL remains poorly defined. In this study, we identified 1326 mutations involving 311 genes or regions in 90 PTL patients through next-generation sequencing (NGS). PTL patients with the TBL1XR1 mutation, irrespective of treatment therapy, had an inferior overall survival (OS) than TBL1XR1 WT (wild type) patients (*p* = 0.045). Moreover, patients with this mutation, treated with a CHOP regimen (CTX 750 mg/m^2^ iv, d1,8 ADM 50 mg/m^2^ iv, d1 VCR 1.4 mg/m^2^ iv, d1 PDN 100 mg/m^2^ po d1–5), had a poorer OS (*p* = 0.019). In addition, such patients were prone to have a more intensive infiltration of tumors (*p* = 0.025, x^2^ = 4.890). Thus, we speculated that patients with a TBL1XR1 mutation have poorer prognosis, partly due to greater invasion and infiltration of tumors. Our results suggest that the TBL1XR1 mutation can be used as an indicator to predict the prognosis of PTL and can be employed as a promising new target for treatment of PTL in the future.

**To the editor:**

Primary testicular lymphoma (PTL) is a rare, clinically aggressive type of extra nodal lymphoma [1]. Approximately 80–98% of PTL cases are diagnosed as diffuse large B-cell lymphoma (DLBCL), a common heterogeneous type of non-Hodgkin’s lymphoma (NHL) [2]. PTL features a high risk of relapse in the central nervous system (CNS) and contralateral testis, directly leading to a poor outcome in the patients [3]. In recent years, the addition of radiotherapy, full-course chemotherapy and CNS-directed prophylaxis and rituximab have greatly improved the prognosis of DLBCL patients; however, the prognosis for PTL remains poor [4]. Previous studies reported that B symptoms, advanced Ann Arbor stage (III/IV), and extra nodal involvement are poor prognostic markers for PTL [5]. MYD88 and CD79B mutations are frequently observed in PTL, but no prognostic impact was observed [6]. The gene mutation landscape and the prognosis of PTL remain poorly defined. In addition, information on different mutations in PTL is not available.

In our study, we used NGS to clarify the mutation landscape of PTL in 90 patients, who attended the First Affiliated Hospital of Soochow University and the First Affiliated Hospital of Nantong University between January 2007 and July 2018. This study was approved by the Ethics committee of the First Affiliated Hospital of Soochow University in accordance to the Declaration of Helsinki. Sixty-six patients (73%) received an anthracyline-based chemotherapy, usually CHOP regimen. Twenty-four patients (27%) were simultaneously treated with rituximab. The median chemotherapy course was six courses. Twelve patients received irradiation aimed at the contralateral testis, and no patient received head irradiation. OS was estimated using the Kaplan-Meier method. The two-sided level of significance was *p* < 0.05. Statistical analyses were performed using SPSS 23.0. The follow-up was updated on August 31, 2019, with a median follow-up time of 36 (1–120) months. Fourteen patients (15.5%) were lost to follow-up (Supplementary Table 1 and 2).

Patients’DNA was extracted from paraffin-embedded tissues in accordance to the manufacturer’s protocol and were sequenced on an Illumina Hiseq 2000 instrument using a targeted panel covering 446 genes (Table 1 in Supplementary Appendix). We identified 1326 mutations involving 311 genes or regions in 90 PTL patients. MYD88 mutations were the most frequently observed mutation, occurring in 75.6% (68/90) patients. Other commonly mutated genes were PIM1 (71.1%), TBL1XR1 (37.8%), KMT2D (37.8%) and KMT2C (34.4%) (Fig. 1a, supplementary information is given in Table 3). There was a positive correlation between TBL1XR1 and PIM1/BTG2 mutations (*r* = 0.244 and *r* = 0.247, respectively) (Table 1). PTL patients with TBL1XR1 mutation, irrespective of treatment therapy, had an inferior OS than TBL1XR1 WT patients (*p* = 0.045, HR 1.854, 95%CI 1.004–3.442) (Fig. 1b). Moreover, patients carrying this mutation, treated with CHOP regimen, also had poorer OS (*p* = 0.019, HR 2.378, 95%CI 1.121–5.045) (Fig. 1c).

**Fig. 1:**
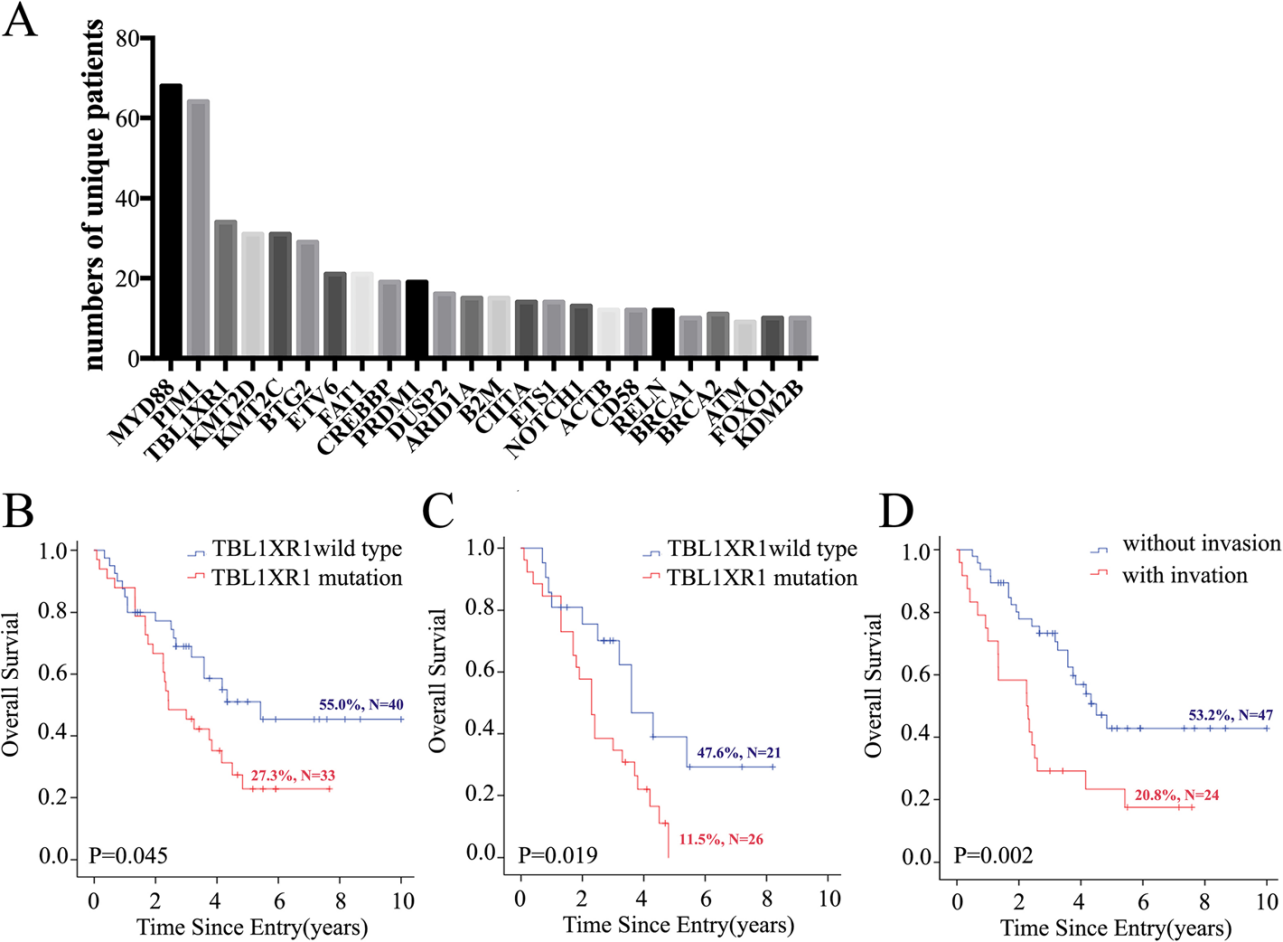
**a** Landscape of Driver Mutations in primary testicular diffuse large B-cell lymphoma; **b** Overall survival rate in all patients with TBL1XR1 mutation or not; **c** Overall survival rate in patients treated with CHOP regimen with TBL1XR1 mutation or not; **d** Overall survival rate in all patients with invasion or not

**Table 1:** Baseline characteristics of the patients

	TBL1XR1 mutation ***N*** = 34	TBL1XR1 WT ***N*** = 56	*P* value
Age (years)^b^	66.5 (46–89)	65 (33–86)	0.25
CD5^a^	4	13	0.178
**Type**^a^			
DLBCL-ABC	27	45	0.913
DLBCL-GCB	3	7	0.737
DLBCL	4	4	0.47
Other	0	3	0.287
**Therapy**^a^			
CHOP	28	39	
R-CHOP	6	17	0.18
**Mutation**^a^			
MYD88	29	39	0.094
PIM1	29	35	0.021*
KMT2D	9	22	0.215
KMT2C	14	17	0.295
BTG2	16	13	0.019*
invasion^a^	11	11	0.027*

^a^Number of patients

^b^Median (range)

*The difference is statistically significant (*p* < 0.05)

TBL1XR1, also known as TBLR1, is an evolutionarily conserved protein that has high structural and functional similarities. It plays an important role in activation of multiple intracellular signaling pathways, such as Wnt-β-catenin, NF-κB, and Notch signaling pathways [7]. Dysregulation of TBL1XR1 has been observed in lots of neoplastic conditions [8]. TBL1XR1 is preferentially expressed in human CD34 + CD38- cells and vital for stem cell balancing. In B-cell acute lymphoblastic leukemia, function loss of TBL1XR1 disrupts glucocorticoid receptor recruitment to chromatin, resulting in glucocorticoid resistance [9].

In addition, patients with TBL1XR1 mutation were prone to have more intensive infiltration of tumors (*p* = 0.025, x^2^ = 4.890). This finding is consistent with a previous study, which reported that abnormal regulation of TBL1XR1 is associated with advanced tumor stage, metastasis, and poor prognosis in most solid tumors [10]. Patients with tumor infiltration had poorer outcomes, and there was a statistical difference between TBL1XR1 mutation and WT groups (*p* = 0.002, HR 2.568, 95%CI 1.382–4.772) (Fig. 1d). OS of patients with TBL1XR1 mutation treated with CHOP regimen was 11.5% whereas OS of patients with TBL1XR1 mutation treated with R-CHOP regimen was 100% (6/6). Thus, we speculate that rituximab may improve the prognosis of patients with TBL1XR1 mutations, but this needs to be further studied by more patients.

In conclusion, we found that TBL1XR1 is commonly mutated in PTL. Patients with TBL1XR1 mutations have lower OS, partly due to greater invasion and infiltration of tumors. Therefore, TBL1XR1 mutation can be used as an indicator to predict the prognosis of PTL and a promising new target for treatment of PTL in future.

## Supplementary information

**Additional file 1:Table S1.** 446 known or putative mutational gene targets in hematologic malignancies detected by the next generation sequencing. **Table S2.** Characteristics of 90 PTL patients. **Table S3.** Mutated characteristics of 34 TBL1XR1 mutation PTL patients

## Data Availability

All data obtained and/or analyzed during the current study were available from the corresponding authors in a reasonable request.

## Abbreviations

PTL: Primary testicular lymphoma
DLBCL: Diffuse large B-cell lymphoma
NHL: Non-Hodgkin’s lymphoma
CNS: Central nervous system
TBL1XR1: Transducin (beta)-like 1X related protein 1
WT: Wild type
OS: Overall survival
CHOP: CTX, ADM, VCR, PDN
