# Supplementary material for: TBL1XR1 mutation predicts poor outcome in primary testicular diffuse large B-cell lymphoma patients
Source: Biomark Res. 2020 Apr 17;8:10. doi: 10.1186/s40364-020-00189-1 (PMC7164167; doi:10.1186/s40364-020-00189-1)
Supplement: Supplementary file 1 — Additional file 1:Table S1. 446 known or putative mutational gene targets in hematologic malignancies detected by the next generation sequencing. Table S2. Characteristics of 90 PTL patients. Table S3. Mutated characteristics of 34 TBL1XR1 mutation PTL patients [file 40364_2020_189_MOESM1_ESM.docx]

**Supplemental Information**

**TBL1XR1 mutation predicts poor outcome in primary testicular diffuse large B-cell lymphoma patients**

Authors: Xinfeng Wang^1,3^ *, Xiaoyu Xu^1,2^ *, Wenzhi Cai^1,2^*, Haiyan Bao^1^, Hongming Huang^3^, Yifei Liu^3^, Xi Yang^3^, Changgeng Ruan ^1,2^, Depei Wu^1,2^ , Hongjie Shen^1 #^, Suning Chen^1,2 #^.

1. Jiangsu Institute of Hematology, Key Laboratory of Thrombosis and Hemostasis of Ministry of Health, the First Affiliated Hospital of Soochow University, Suzhou, P.R. China.
2. Institute of Blood and Marrow Transplantation, Collaborative Innovation Center of Hematology, Soochow University, Suzhou, P.R. China.
3. The First Affiliated Hospital of Nantong University, Nantong, P.R. China.

**Materials and Methods**

**DNA isolation**

Genomic DNA was isolated from primary tumor tissue using PureLink Genomic DNA Mini Kit (Qiagen) according to the manufacturer’s instructions.

**Targeted sequencing**

Targeted sequencing of the entire coding sequences of 446 known or putative mutational gene targets in hematologic malignancies was performed on 90 PTL patients. Briefly, the genomic DNA was sheared and the sample libraries prepared using the TruSeq DNA Sample Preparation Kit (Illumina, San Diego, CA). Sequencing was performed using a custom amplicon-based targeted enrichment assay (Haloplex, Agilent, Boeblingen, Germany) and an Illumina MiSeq instrument (Illumina).

**Statistical analysis**

Statistical analyses were performed using the software SPSS 23.0 and Graph Pad Prism 7. Survival probabilities were estimated by the Kaplan-Meier method. Differences in categorical variables were calculated using a chi-square test and differences in continuous variables by means of the Mann-Whitney U-test. For all analyses, P values were two-sided, and P<0.05 was considered statistically significant.

**Supplementary Table 1. 446 known or putative mutational gene targets in hematologic malignancies detected by the next generation sequencing**

| ABCB1 | AURKA | BTK | CDK4 | CYP2A6 | EPCAM |
| --- | --- | --- | --- | --- | --- |
| ABCB4 | AURKB | BTLA | CDK6 | CYP2A7 | EPHA2 |
| ABCC2 | AXIN1 | BUB1B | CDK8 | CYP2B6 | EPHA3 |
| ABL1 | AXL | CALR | CDKN1B | CYP2C19 | ERBB2 |
| ABL2 | B2M | CARD11 | CDKN1C | CYP2C9 | ERBB3 |
| ACTA1 | BAP1 | CBFB | CDKN2A | CYP2D6 | ERBB4 |
| ACTB | BARD1 | CBL | CDKN2B | CYP3A4 | ERCC1 |
| ADH1B | BCL10 | CBLB | CDKN2C | CYP3A5 | ERCC2 |
| AIM1 | BCL11B | CCND1 | CEBPA | DAXX | ERCC3 |
| AIP | BCL2 | CCND2 | CEP57 | DDR2 | ERCC4 |
| AKT1 | BCL2L1 | CCND3 | CHD8 | DDX3X | ERCC5 |
| AKT2 | BCL2L11 | CCNE1 | CHEK1 | DHFR | ERG |
| AKT3 | BCL2L2 | CCT6B | CHEK2 | DICER1 | ESR1 |
| ALDH2 | BCL6 | CD22 | CIITA | DNM2 | ETS1 |
| ALK | BCL7A | CD274 | CKS1B | DNMT3A | ETV1 |
| AP3B1 | BCOR | CD28 | CREBBP | DNMT3B | ETV4 |
| APC | BCORL1 | CD58 | CRLF2 | DOT1L | ETV6 |
| AR | BCR | CD70 | CSF1R | DPYD | EWSR1 |
| ARHGAP26 | BIRC3 | CD74 | CSF3R | DTX1 | EZH2 |
| ARID1A | BLM | CD79A | CTCF | DUSP2 | FANCA |
| ARID1B | BMPR1A | CD79B | CTLA4 | EBF1 | FANCC |
| ARID2 | BRAF | CD83 | CTNNB1 | ECSIT | FANCD2 |
| ARID5B | BRCA1 | CDA | CUX1 | ECT2L | FANCE |
| ASXL1 | BRCA2 | CDC73 | CXCR4 | EED | FANCF |
| ATM | BRD4 | CDH1 | CYLD | EGFR | FANCG |
| ATR | BRIP1 | CDK10 | CYP19A1 | EGR1 | FANCL |
| ATRX | BTG2 | CDK12 | CYP2A13 | EP300 | FAS |
| FAT1 | HBB | KDR | MITF | NQO1 | PMS2 |
| FBXO11 | HDAC1 | KIT | MLH1 | NRAS | POLE |
| FBXW7 | HDAC2 | KLHL6 | MLH3 | NSD1 | POT1 |
| FGFR1 | HDAC4 | KLLN | MPL | NT5C2 | PPP2R1A |
| FGFR2 | HDAC7 | KMT2A | MRE11A | NTRK1 | PRDM1 |
| FGFR3 | HGF | KMT2B | MSH2 | NTRK3 | PRF1 |
| FGFR4 | HNF1A | KMT2C | MSH3 | NUP98 | PRKAR1A |
| FH | HNF1B | KMT2D | MSH6 | P2RY8 | PTCH1 |
| FIP1L1 | HRAS | KRAS | MTHFR | PAG1 | PTEN |
| FLCN | ID3 | LAMP1 | MTOR | PAK3 | PTPN11 |
| FLT1 | IDH1 | LEF1 | MUTYH | PALB2 | PTPN13 |
| FLT3 | IDH2 | LMO1 | MYC | PAX5 | PTPN2 |
| FLT4 | IGF1R | LMO2 | MYCL | PBRM1 | PTPN6 |
| FOXO1 | IKBKE | LYN | MYCN | PC | PTPRD |
| FOXO3 | IKZF1 | LYST | MYD88 | PDCD1 | PTPRO |
| GADD45B | IKZF2 | MAF | MYH11 | PDCD1LG2 | RAB27A |
| GATA1 | IKZF3 | MAFB | NAT1 | PDE11A | RAC3 |
| GATA2 | IL7R | MALT1 | NBN | PDGFRA | RAD21 |
| GATA3 | INPP4B | MAP2K1 | NCSTN | PDGFRB | RAD50 |
| GNA11 | INPP5D | MAP2K2 | NF1 | PDK1 | RAD51 |
| GNA13 | IRF1 | MAP2K4 | NF2 | PGR | RAF1 |
| GNAQ | IRF4 | MAP3K1 | NFKB1 | PHF6 | RARA |
| GNAS | IRF8 | MAP3K14 | NFKB2 | PHOX2B | RASGEF1A |
| GRIN2A | JAK1 | MCL1 | NFKBIA | PIK3CA | RB1 |
| GSTM1 | JAK2 | MDM2 | NFKBIE | PIK3CD | RECQL4 |
| GSTM4 | JAK3 | MDM4 | NKX2-1 | PIK3R1 | RELN |
| GSTM5 | JARID2 | MED12 | NKX2-2 | PIK3R2 | RET |
| GSTP1 | JUN | MEF2B | NKX2-4 | PIM1 | RHOA |
| GSTT1 | KDM2B | MEN1 | NOTCH1 | PLCG2 | RICTOR |
| HBA1 | KDM5A | MET | NOTCH2 | PML | RNF43 |
| HBA2 | KDM6A | MGMT | NPM1 | PMS1 | ROS1 |
| RPTOR | SGK1 | SPOP | TBL1XR1 | TOP2A | UGT1A1 |
| RRM1 | SH2D1A | SRC | TCF3 | TP53 | UNC13D |
| RUNX1 | SLC34A2 | SRSF2 | TCL1A | TP63 | VEGFA |
| RUNX1T1 | SMAD2 | STAG2 | TEK | TP73 | VHL |
| SBDS | SMAD4 | STAT3 | TEKT4 | TPMT | WHSC1 |
| SDC4 | SMAD7 | STAT5A | TERT | TRAF2 | WT1 |
| SDHA | SMARCA4 | STAT5B | TET2 | TRAF3 | XIAP |
| SDHAP1 | SMARCB1 | STAT6 | TGFBR2 | TRAF5 | XPC |
| SDHAP2 | SMC1A | STIL | TLE1 | TSC1 | XPO1 |
| SDHAP3 | SMC3 | STK11 | TLE4 | TSC2 | XRCC1 |
| SDHB | SMO | STMN1 | TMPRSS2 | TSHR | YAP1 |
| SDHC | SOCS1 | STX11 | TNFAIP3 | TTF1 | ZAP70 |
| SDHD | SOX1 | STXBP2 | TNFRSF11A | TUBB2A | ZRSR2 |
| SERP2 | SOX14 | SUFU | TNFRSF14 | TUBB2B |  |
| SETBP1 | SOX2 | SUZ12 | TNFRSF17 | TUBB3 |  |
| SETD2 | SOX21 | SYK | TNFRSF19 | TYMS |  |
| SF3B1 | SPEN | TAL1 | TOP1 | U2AF1 |  |

**Supplementary Table 2 Characteristics of 90 PTL patients**

| **number** | **age** | **CD5** | **diagnosis** | **therapy** | **Chemotherapy course** | **infiltration** | **radiation** | **status** | **diagnosed time** | **dead time** |
| --- | --- | --- | --- | --- | --- | --- | --- | --- | --- | --- |
| 1 | 49 | 0 | DLBCL（ABC） | CHOP | 4 | 0 | 0 | 0 | 2012/1/1 | 2019/9/1 |
| 2 | 67 | 0 | DLBCL（ABC） | R-CHOP | 3 | 0 | 1 | 0 | 2015/5/1 | 2019/9/1 |
| 3 | 81 | 0 | DLBCL（ABC） | CHOP | 4 | 0 | 0 | 1 | 2018/6/1 | 2018/12/1 |
| 4 | 59 | 0 | DLBCL（ABC） | CHOP | 5 | 0 | 0 | 0 | 2018/5/1 | 2019/9/1 |
| 5 | 65 | 0 | DLBCL（GCB） | R-CHOP | 6 | 0 | 0 | 0 | 2018/4/1 | 2019/9/1 |
| 6 | 51 | 0 | DLBCL（GCB） | R-CHOP | 4 | 0 | 1 | 0 | 2016/7/1 | 2019/9/1 |
| 7 | 67 | 1 | DLBCL（ABC） | CHOP | 4 | 0 | 0 | 1 | NA | NA |
| 8 | 66 | 0 | DLBCL（ABC） | R-CHOP | 6 | 1 | 1 | 0 | 2012/2/1 | 2019/9/1 |
| 9 | 55 | 0 | DLBCL（ABC） | CHOP | 6 | 1 | 0 | 1 | 2013/6/1 | 2017/7/28 |
| 10 | 62 | 1 | DLBCL（ABC） | R-CHOP | 6 | 0 | 0 | 1 | 2014/6/1 | 2018/8/1 |
| 11 | 69 | 0 | DLBCL（GCB） | CHOP | 6 | 0 | 0 | NA | NA | NA |
| 12 | 77 | 0 | DLBCL（ABC） | R-CHOP | 6 | 0 | 0 | 0 | 2015/12/1 | 2019/9/1 |
| 13 | 66 | 1 | DLBCL（GCB） | R-CHOP | 6 | 0 | 0 | 0 | 2014/7/1 | 2019/9/1 |
| 14 | 60 | 1 | DLBCL（ABC） | NA | 4 | 0 | 0 | 1 | NA | NA |
| 15 | 74 | 0 | DLBCL（ABC） | CHOP | 6 | 0 | 0 | 1 | 2016/4/1 | 2018/9/1 |
| 16 | 49 | 0 | DLBCL（ABC） | CHOP | 6 | 0 | 0 | 0 | 2014/3/1 | 2019/9/1 |
| 17 | 76 | 0 | DLBCL（ABC） | CHOP | 2 | 1 | 0 | 1 | 2013/4/1 | 2013/12/1 |
| 18 | 67 | 0 | DLBCL（ABC） | CHOP | 4 | 1 | 0 | 1 | 2015/5/1 | 2017/10/1 |
| 19 | 75 | 0 | DLBCL（ABC） | CHOP | 6 | 0 | 0 | 0 | 2011/7/1 | 2019/9/1 |
| 20 | 57 | 0 | DLBCL（ABC） | CHOP | 8 | 0 | 0 | 0 | 2015/5/1 | 2019/9/1 |
| 21 | 77 | 0 | DLBCL（ABC） | CHOP | 4 | 0 | 0 | 1 | 2015/9/1 | 2019/4/1 |
| 22 | 68 | 0 | DLBCL（ABC） | CHOP | 4 | 1 | 0 | 1 | 2012/11/1 | 2015/2/1 |
| 23 | 80 | 0 | DLBCL（ABC） | R-CHOP | 4 | 0 | 0 | 0 | 2016/10/1 | 2019/9/1 |
| 24 | 84 | 0 | DLBCL（ABC） | CHOP | 2 | 1 | 0 | 0 | 2016/9/1 | 2019/9/1 |
| 25 | 60 | 1 | DLBCL（ABC） | R-CHOP | 6 | 0 | 0 | 1 | 2012/10/1 | 2015/6/1 |
| 26 | 67 | 0 | DLBCL（ABC） | R-CHOP | 4 | 0 | 0 | 0 | 2013/10/1 | 2019/9/1 |
| 27 | 42 | 1 | DLBCL（GCB） | CHOP | 4 | 1 | 0 | 0 | 2012/7/1 | 2019/9/1 |
| 28 | 83 | 0 | DLBCL（ABC） | CHOP | 6 | 0 | 0 | 1 | 2013/7/1 | 2015/6/1 |
| 29 | 67 | 1 | DLBCL（ABC） | CHOP | 4 | 1 | 1 | 1 | 2016/8/1 | 2017/1/1 |
| 30 | 75 | 0 | DLBCL | CHOP | 2 | 1 | 0 | 1 | 2012/7/1 | 2012/9/1 |
| 31 | 48 | 0 | DLBCL（ABC） | R-CHOP | 6 | 0 | 0 | 0 | 2012/5/1 | 2019/9/1 |
| 32 | 46 | 0 | DLBCL（ABC） | R-CHOP | 3 | 1 | 0 | 0 | 2014/3/1 | 2019/9/1 |
| 33 | 88 | 1 | DLBCL（ABC） | CHOP | 2 | 0 | 0 | 1 | 2014/10/1 | 2018/7/1 |
| 34 | 67 | 0 | DLBCL（ABC） | R-CHOP | 5 | 0 | 0 | 0 | 2013/10/1 | 2019/9/1 |
| 35 | 65 | 0 | DLBCL（ABC） | CHOP | 1 | 1 | 0 | 1 | 2016/6/1 | 2016/7/1 |
| 36 | 86 | 0 | DLBCL（ABC） | CHOP | 2 | 1 | 0 | 1 | 2014/3/1 | 2015/3/1 |
| 37 | 65 | 0 | DLBCL | R-CHOP | 8 | 0 | 0 | 0 | 2011/1/1 | 2019/9/1 |
| 38 | 55 | 0 | DLBCL（ABC） | R-CHOP | 7 | 0 | 1 | 0 | 2009/9/1 | 2019/9/1 |
| 39 | 77 | 0 | DLBCL（ABC） | R-CHOP | 3 | 1 | 0 | 1 | 2012/6/1 | 2012/10/1 |
| 40 | 71 | 1 | DLBCL（ABC） | CHOP | 4 | 0 | 0 | 1 | 2013/3/1 | 2013/12/1 |
| 41 | 68 | 1 | DLBCL（GCB） | CHOP | 5 | 0 | 0 | 1 | 2010/12/1 | 2014/2/1 |
| 42 | 58 | 1 | DLBCL（ABC） | CHOP | 6 | 1 | 0 | 1 | 2011/8/1 | 2017/1/1 |
| 43 | 83 | 0 | DLBCL | CHOP | 6 | 0 | 0 | NA | NA | NA |
| 44 | 63 | 0 | DLBCL（ABC） | R-CHOP | 6 | 0 | 0 | 0 | 2014/9/1 | 2019/9/1 |
| 45 | 51 | 0 | DLBCL（ABC） | R-CHOP | 6 | 0 | 0 | 0 | 2015/7/1 | 2019/9/1 |
| 46 | 84 | 0 | DLBCL（ABC） | CHOP | 4 | 1 | 0 | 1 | 2014/11/1 | 2016/3/1 |
| 47 | 78 | 0 | DLBCL（ABC） | CHOP | 4 | 1 | 0 | 1 | 2015/10/1 | 2016/9/1 |
| 48 | 87 | 0 | DLBCL（ABC） | CHOP | 4 | 1 | 0 | 1 | 2016/4/1 | 2017/8/1 |
| 49 | 57 | 0 | DLBCL（ABC） | R-CHOP | 6 | 1 | 1 | 1 | 28months | NA |
| 50 | 57 | 0 | DLBCL（ABC） | NA | 6 | 1 | 0 | 1 | 2014/5/1 | 2016/12/1 |
| 51 | 89 | 0 | DLBCL（ABC） | CHOP | 2 | 0 | 0 | 1 | 2014/5/1 | 2016/2/1 |
| 52 | 65 | 0 | DLBCL（ABC） | CHOP | 6 | 0 | 0 | NA | NA | NA |
| 53 | 71 | 0 | DLBCL（ABC） | CHOP | 6 | 0 | 0 | NA | NA | NA |
| 54 | 62 | 0 | DLBCL | CHOP | 4 | 1 | 0 | 0 | 2016/4/1 | 2019/9/1 |
| 55 | 71 | 1 | DLBCL（ABC） | CHOP | 4 | 0 | 0 | 1 | 2018/4/1 | NA |
| 56 | 72 | 0 | DLBCL（ABC） | CHOP | 4 | 0 | 0 | 1 | 2007/1/1 | 2011/5/1 |
| 57 | 57 | 0 | DLBCL（ABC） | CHOP | 6 | 0 | 1 | 1 | 2008/12/1 | 2012/7/1 |
| 58 | 60 | 0 | DLBCL（ABC） | CHOP | 5 | 0 | 0 | 0 | 2015/8/1 | 2019/9/1 |
| 59 | 67 | 1 | DLBCL（GCB） | CHOP | 6 | 1 | 0 | 1 | 2015/7/1 | 2018/1/1 |
| 60 | 0 | 0 | DLBCL（ABC） | CHOP | 6 | 0 | 0 | NA | NA | NA |
| 61 | 62 | 0 | DLBCL（ABC） | CHOP | 12 | 0 | 1 | 1 | 2012/12/1 | 2013/8/1 |
| 62 | 62 | 0 | DLBCL（ABC） | CHOP | 6 | 0 | 0 | 1 | 2014/5/1 | 2017/8/1 |
| 63 | 65 | 0 | DLBCL（ABC） | CHOP | 2 | 0 | 0 | 1 | 2017/8/1 | 2019/4/1 |
| 64 | 61 | 0 | DLBCL（ABC） | CHOP | 4 | 0 | 1 | 0 | 2017/1/1 | 2019/9/1 |
| 65 | 66 | 1 | DLBCL（ABC） | CHOP | 6 | 1 | 0 | 1 | 2014/4/1 | 2016/8/1 |
| 66 | 60 | 0 | DLBCL（ABC） | CHOP | 6 | 1 | 0 | 1 | 2014/5/1 | 2016/8/1 |
| 67 | 79 | 0 | DLBCL（GCB） | CHOP | 6 | 0 | 0 | 1 | 2018/1/1 | NA |
| 68 | 52 | 0 | DLBCL（ABC） | CHOP | 4 | 0 | 0 | 1 | NA | NA |
| 69 | 58 | 1 | DLBCL（ABC） | CHOP | 6 | 0 | 0 | 1 | 2008/5/1 | 2010/5/1 |
| 70 | 70 | 0 | DLBCL（ABC） | CHOP | 4 | 0 | 0 | 1 | NA | NA |
| 71 | 62 | 0 | DLBCL（ABC） | CHOP | 4 | 0 | 0 | 1 | 2009/1/1 | NA |
| 72 | 64 | 1 | DLBCL（ABC） | R-CHOP | 10 | 0 | 0 | 1 | 2012/1/1 | 2013/2/1 |
| 73 | 66 | 0 | DLBCL（ABC） | R-CHOP | 6 | 0 | 1 | 0 | 2016/8/1 | 2019/9/1 |
| 74 | 71 | 0 | DLBCL（ABC） | CHOP | 6 | 0 | 0 | 1 | 2014/5/1 | 2019/3/1 |
| 75 | 65 | 0 | DLBCL（ABC） | R-CHOP | 5 | 0 | 0 | 0 | 2015/1/1 | 2019/9/1 |
| 76 | 83 | 0 | DLBCL（ABC） | CHOP | 6 | 0 | 0 | 0 | 2015/1/1 | 2019/9/1 |
| 77 | 74 | 0 | DLBCL（ABC） | CHOP | 4 | 1 | 0 | 1 | 2017/2/1 | 2018/6/1 |
| 78 | 47 | 0 | DLBCL（GCB） | CHOP | 6 | 0 | 0 | 0 | 2017/1/1 | 2019/9/1 |
| 79 | 76 | 0 | DLBCL（ABC） | CHOP | 6 | 0 | 1 | 1 | 2013/12/1 | 2018/6/1 |
| 80 | 70 | 1 | DLBCL（ABC） | R-CHOP | 6 | 0 | 0 | 1 | 2017/11/1 | 2018/12/1 |
| 81 | 66 | 0 | DLBCL（GCB） | CHOP | 6 | 0 | 0 | 1 | 2015/12/1 | 2018/12/1 |
| 82 | 50 | 0 | DLBCL（ABC） | R-CHOP | 6 | 0 | 1 | 0 | 2013/10/1 | 2019/9/1 |
| 83 | 77 | 0 | DLBCL（ABC） | CHOP | 6 | 0 | 0 | 1 | 2014/4/1 | 2015/12/1 |
| 84 | 83 | 0 | DLBCL | CHOP | 6 | 0 | 0 | 1 | 2012/1/1 | 2015/11/1 |
| 85 | 57 | 0 | Peripheral T cell lymphoma | CHOP | 4 | 1 | 0 | 1 | NA | NA |
| 86 | 53 | 0 | Marginal B-cell lymphoma | CHOP | 6 | 0 | 0 | 0 | 2018/3/1 | 2019/9/1 |
| 87 | 33 | 0 | B lymphoblastic lymphoma | CHOP | 4 | 0 | 0 | 1 | NA | NA |
| 88 | NA | 0 | DLBCL | CHOP | 6 | 0 | 0 | NA | NA | NA |
| 89 | NA | 0 | DLBCL | CHOP | 6 | 0 | 0 | NA | NA | NA |
| 90 | NA | 0 | DLBCL | CHOP | 6 | 0 | 0 | NA | NA | NA |

**Supplementary Table 3 Mutated characteristics of 34 TBL1XR1 mutation PTL patients**

|  | **Gene.ID** | **AA.Change** | **COSMIC recurrence** | | **dbSNP** |
| --- | --- | --- | --- | --- | --- |
| 1 | TBL1XR1:NM_024665.4:exon14 | p.Y446C (c.A1337G) | 2 | missense_variant | |
| 6 | TBL1XR1:NM_024665.4:exon13 | p.H390R (c.A1169G) | 1 | missense_variant | |
| 9 | TBL1XR1:NM_024665.4:exon14 | p.D421E (c.T1263A) | 0 | missense_variant | |
| 13 | TBL1XR1:NM_024665.4:exon11 | p.H348L (c.A1043T) | 0 | missense_variant | |
| 15 | TBL1XR1:NM_024665.4:exon13 | p.I394F (c.A1180T) | 0 | missense_variant | |
| 18 | TBL1XR1:NM_024665.4:exon13 | p.E393V (c.A1178T) | 0 | missense_variant | |
| 18 | TBL1XR1:NM_024665.4:exon5 | p.K102fs (c.305delA) | 0 | frameshift_variant | |
| 22 | TBL1XR1:NM_024665.4:exon14 | p.441_442del (c.1321_1326delCACCAA) | 0 | inframe_deletion | |
| 26 | TBL1XR1:NM_024665.4:exon14 | p.S419F (c.C1256T) | 4 | missense_variant | |
| 28 | TBL1XR1:NM_024665.4:exon14 | p.Y446C (c.A1337G) | 2 | missense_variant | |
| 29 | TBL1XR1:NM_024665.4:exon13 | p.I394M (c.T1182G) | 0 | missense_variant | |
| 30 | TBL1XR1:NM_024665.4:exon9 | p.H265Y (c.C793T) | 3 | missense_variant | |
| 32 | TBL1XR1:NM_024665.4:exon11 | p.324_325del (c.971_973delCTT) | 4 | inframe_deletion | |
| 32 | TBL1XR1:NM_024665.4:exon11 | p.H348L (c.A1043T) | 0 | missense_variant | |
| 33 | TBL1XR1:NM_024665.4:exon12 | p.S366P (c.T1096C) | 2 | missense_variant | |
| 35 | TBL1XR1:NM_024665.4:exon13 | p.H390R (c.A1169G) | 1 | missense_variant | |
| 46 | TBL1XR1:NM_024665.4:exon13 | p.Y395C (c.A1184G) | 1 | missense_variant | |
| 48 | TBL1XR1:NM_024665.4:exon6 | p.C175F (c.G524T) | 0 | missense_variant | |
| 49 | TBL1XR1:NM_024665.4:exon13 | p.Y395C (c.A1184G) | 1 | missense_variant | |
| 51 | TBL1XR1:NM_024665.4:exon14 | p.A418T (c.G1252A) | 0 | missense_variant;splice_region_variant | |
| 51 | TBL1XR1:NM_024665.4:exon12 | p.S366P (c.T1096C) | 2 | missense_variant | |
| 51 | TBL1XR1:NM_024665.4:exon11 | p.T327P (c.A979C) | 0 | missense_variant | |
| 54 | TBL1XR1:NM_024665.4:exon13 | p.Y395C (c.A1184G) | 1 | missense_variant | |
| 58 | TBL1XR1:NM_024665.4:exon9 | p.G267C (c.G799T) | 1 | missense_variant | |
| 61 | TBL1XR1:NM_024665.4:exon12 | p.E351A (c.A1052C) | 0 | missense_variant | |
| 62 | TBL1XR1:NM_024665.4:exon14 | p.S419F (c.C1256T) | 4 | missense_variant | |
| 63 | TBL1XR1:NM_024665.4:exon11 | p.C325F (c.G974T) | 2 | missense_variant | |
| 65 | TBL1XR1:NM_024665.4:exon10 | p.H307R (c.A920G) | 4 | missense_variant | |
| 66 | TBL1XR1:NM_024665.4:exon14 | p.S419P (c.T1255C) | 1 | missense_variant | |
| 74 | TBL1XR1:NM_024665.4:exon14 | p.S461Y (c.C1382A) | 0 | missense_variant | |
| 76 | TBL1XR1:NM_024665.4:exon13 | p.Y395C (c.A1184G) | 1 | missense_variant | |
| 77 | TBL1XR1:NM_024665.4:exon14 | p.F420S (c.T1259C) | 0 | missense_variant | |
| 77 | TBL1XR1:NM_024665.4:exon16 | p.L510S (c.T1529C) | 0 | missense_variant | |
| 79 | TBL1XR1:NM_024665.4:exon6 | p.L184Q (c.T551A) | 0 | missense_variant | |
| 81 | TBL1XR1:NM_024665.4:exon14 | p.H441Y (c.C1321T) | 0 | missense_variant | |
| 82 | TBL1XR1:NM_024665.4:exon8 | p.253fs (c.759_760insTTAT) | 0 | frameshift_variant | |
| 83 | TBL1XR1:NM_024665.4:exon13 | p.Y395C (c.A1184G) | 1 | missense_variant | |
| 84 | TBL1XR1:NM_024665.4:exon5 | p.S123Y (c.C368A) | 0 | missense_variant | |
| 84 | TBL1XR1:NM_024665.4:exon5 | p.S123fs (c.369delT) | 0 | frameshift_variant | |
| 90 | TBL1XR1:NM_024665.4:exon9 | p.G285E (c.G854A) | 0 | missense_variant | |
